# Post-exposure Period as a key Factor to Assess Cadmium Toxicity: Lethal vs. Behavioral Responses

**DOI:** 10.1007/s00128-022-03651-y

**Published:** 2023-01-18

**Authors:** Álvaro Alonso

**Affiliations:** Universidad de Alcalá, Facultad de Ciencias, Departamento de Ciencias de la Vida, Unidad Docente de Ecología, Biological Invasions Research Group, Universidad de Alcalá, Plaza San Diego s/n, 28801 Alcalá de Henares, Spain

**Keywords:** Behavioral activity, Cadmium, Recovery, Mortality

## Abstract

The exposure of animals to pollution in ecosystems is not always chronic. Toxicants can remain in aquatic ecosystems for a short-term. To improve the extrapolation of laboratory results to natural scenarios the inclusion of post-exposure periods is a relevant issue. The present study focuses on the assessment of cadmium toxicity on survival and behavior of the aquatic snail *Potamopyrgus antipodarum* (Tateidae, Mollusca) during exposure and post-exposure. Animals were exposed for 48 h to cadmium (0.05, 0.14, 0.44 and 1.34 mg Cd/L) and 168 h of post-exposure. During the post-exposure period an increase in mortality in all concentrations was observed. The effects observed during the post-exposure period on the LC50 and EC50 were significant. During the post-exposure, behavior showed a clear recovery in surviving animals exposed to 0.44 mg Cd/L. Animals exposed to 0.05 mg Cd/L did not show differences with control. Therefore, mortality after exposure should be included in the ecotoxicological bioassays for a more realistic estimation of the cadmium effects. To assess the degree of animal recovery after cadmium exposure, behaviour has been shown as an adequate parameter.

In natural ecosystems, the exposure of animals to pollution is not always chronic. Some toxicants can be in aquatic ecosystems during a short period, from a few hours to a few days (Handy 1994; Brent and Herricks 1998; Bundschuh et al. 2013; Zhao and Newman 2006; Kanu et al. 2022). Therefore, animals may face toxicants for a brief period and subsequently they could recover or not during the post-exposure period, which depends on the chemical compound, duration of the exposure and concentration of the toxicant (Zhao and Newman 2006; Hoang et al. 2007; Alonso and Camargo 2009; Lahman and Moore 2015; Kanu et al. 2022). This post-exposure period can help animals to recover fitness if detoxification processes are efficient (Handy 1994; Schill et al. 2003; Zhao and Newman 2006). However, there may be a worsening of animals if toxic threshold has been surpassed during exposure (Handy 1994; Zhao and Newman 2006; Bundschuh et al. 2013; Kanu et al. 2022). Therefore, to improve the extrapolation of laboratory results to natural ecosystems the inclusion of post-exposure periods in experimental setups is a relevant issue in ecotoxicology.

Among ecotoxicological endpoints, mortality is amply used in laboratory bioassays because its utility for sensitivity comparison between species, and it is usually monitored at short-term and with low-cost. Consequently, most of the data used for environmental risk assessment are based on lethal parameters (Constable et al. 2003; Romero-Blanco and Alonso 2022; Wang et al. 2022). Yet, other parameters, such as reproduction, development, and behavior present obvious advantages. For instance, behavioral bioassays are more sensitive and require lower toxicant concentrations than lethal bioassays (Melvin and Wilson 2013). In fact, behavioral changes are the first response to stress at individual scale (Hellou 2011; Melvin and Wilson 2013; Alonso 2021). Therefore, behavior alteration is an early signal of toxicant exposure (Hellou 2011). Moreover, the recovery of previous behavior to toxicant exposure at the end of the post-exposure period may be a good indicator of animal recovery.

Among toxicants, cadmium has been amply studied in ecotoxicology (Wright and Welbourn 1994; Irfan et al. 2021). Cadmium is a non-essential metal, which can be toxic at very low concentrations (Wright and Welbourn 1994). There are several evidence that this metal cause impartments in behavior for different aquatic species, including vertebrates and invertebrates (Bryan et al. 1995; Sornom et al. 2012; Alonso and Valle-Torres 2018). Therefore, it is an appropriate model toxicant in behavioral studies. Among behavioral variables, movement has important ecological implications given that it is involved in most interactions between animals and their environment, including escaping from polluted areas or locating food (Alonso and Valle-Torres 2018; Araujo and Blasco 2019). Therefore, a fast recovery of movements after toxicant exposure may imply a greater probability of survival under natural conditions.

The aim of the present study is to assess the effects of cadmium on survival and behavioral activity of the aquatic snail *Potamopyrgus antipodarum* (Tateidae, Mollusca) during exposure and post-exposure periods. We expect an increase of mortality in the post-exposure period, and a recovery of activity in surviving animals. Additionally, toxic effects of cadmium during exposure and post-exposure periods will be compared to elucidate the contribution of post-exposure period on the adverse effects of cadmium.

## Materials and methods

A culture of *P. antipodarum* was used as a source of animals for the bioassay. The culture was started in 2009 with animals collected in the upper reach of the Henares River (Guadalajara, Spain) and reared at the University of Alcalá (Laboratory of Ecology, Department of Life Sciences). Standardized USEPA water (96 mg NaHCO_3_, 60 mg CaSO_4_·2H_2_O, 4 mg KCl, 122.2 mg MgSO_4_, per litre of deionized water plus 10 mg CaCO_3_ per litre) is used for the culture (USEPA 2002). Snails are reared in 60 L aquaria. 0.10 mg of dry food per animal and day are provided (50% fish food Tetra- Menü© GmbH, Melle, Germany + 50% Sera© Spirulina Tabs GmbH, Heinsberg, Germany). Every two weeks, 10% of the water is renewed. Aquarium water is filtered by means of waterfall filter, which also provides water aeration. This filter physically traps particles in the water by pushing water through filter materials and the filtered water falls into the aquarium.

Before exposure to cadmium, 120 adult animals were taken from the culture and situated randomly in two aquaria (1 L) (60 animals per aquarium). Aquaria were previously filled with 1 L of standardized USEPA water. Aquaria were kept during 7 days at 18ºC (climatic chamber ANSONIC). In the second day of acclimatization, animals were fed with the same food as the cultures. After two hours water was renewed. This was repeated for the two batches (see below). No animals died during this period.

Animals were exposed to four concentrations of cadmium (nominal concentrations of 0.05, 0.12, 0.5 and 1.5 mg Cd/L) and control. These concentrations were based on previous study on the cadmium toxicity to this species (Alonso and Valle-Torres 2018). The cadmium exposure was conducted in two batches. In the first one, control and the two lowest cadmium concentrations were used. In the second batch, control and the two highest cadmium concentrations were used. For both batches, cadmium solutions were prepared from a stock solution of cadmium chloride (80.06 mg CdCl_2_/100 mL) (SIGMA ALDRICH 655198-5G MKBB2360, purity of 99.99% Steinheim, Germany). Animals were exposed to cadmium for 48 h; subsequently, surviving organisms were transferred to control water (USEPA water) and kept for 7 days of post-exposure period. Therefore, the bioassay lasted for 9 days. Thirty replicates were used in each cadmium treatment. In each replicate, an animal was placed in a glass vessel of 30 ml. Sixty animals were used for the control.

Three variables were monitored during the bioassay: mortality, immobility, and time to start activity. An animal was considered as dead if no reaction was observed when the operculum of an inactive animals was touched with forceps. Immobility and the time to start activity (in seconds) were considered as behavioral variables. The time to start activity was the time spent by animals to start the sliding movement (Alonso 2021). This variable was individually monitored, taking each snail up with forceps and placing it in the centre of the vessel, with the operculum facing to the bottom. The time to start activity was recorded by means of a chronometer (Alonso 2021). If after 150 s the animal did not move, it was considered as immobile (Romero-Blanco et al. 2021). Proportioning of mortality and immobility were monitored after 24 and 48 h of cadmium exposure, and after 24, 48, 120 and 168 h of post-exposure to cadmium. Time to start activity was monitored at 0, 24 and 48 h of cadmium exposure, and at 24, 48, 120 and 168 h of post-exposure to cadmium. A stereomicroscope (MOTIC® SMZ-168) equipped with optic fiber beam (Jenalux® 150) was used for the monitoring of variables.

During the bioassay, water temperature (ºC), conductivity (microS/cm), dissolved oxygen (mg O_2_/l) and pH were monitored. An oximeter (Crison® Oxi 45+), conductivimeter (for conductivity and water temperature) (Crison® CM35+) and pHmeter (Crison micropH 2001, ALELLA 08328) were used. Actual cadmium concentrations were monitored at 0 and 48 h of exposure through a spectrophotometer (Spectroquant© NOVA60, Merck, KGaA, 64,293 Darmstadt, Germany) and the Spectroquant Cadmium Test (1.01745.0001, Spectroquant©, Merck, KGaA, 64,271 Darmstadt, Germany). Spectroquant© method has a sensitivity ranging from 0.002 to 0.5 mg Cd/L. Samples of the two highest concentrations were diluted before cadmium analysis. This method is based on the reaction of cadmium ions with 1-(4-nitrophenyl)-3-(4-phenylazophenyl)triazene). The analytical quality assurance of the method was checked following the recommendations of Spectroquant Cadmium Test (1.01745.0001, Spectroquant, Merck©). Six randomly selected replicates of each treatment were used to measure the actual cadmium concentrations. After 48 h of post-exposure, water was renewed. Before water renovation, animals were fed with the same food as the cultures. At the end of the bioassay, seven animals of each treatment were randomly selected to measure the shell length using a micrometer installed in the stereomicroscope (MOTIC® SMZ-168).

To assess the effects of cadmium on mortality and on mortality plus immobility, the LC50 and EC50 were calculated, respectively. The cumulative mortality at 48 h of cadmium exposure and at 168 h of post-exposure were used to calculate the LC50 and their 95% confidence intervals. The cumulative mortality plus immobility were also used at the same exposure and post-exposure periods. Actual cadmium concentrations were used to calculate LC and EC values. To assess if post-exposure period has any influence on cadmium sensitivity, the statistical differences between LC50 48 h and LC50 168 h (and between EC50 48 h and EC50 168 h) were assessed by means of an overlap test or a Z test (Wheeler et al. 2006). If the 95% confidence intervals do not overlap, the LC or EC values were statistically different (p < 0.05). LC50 and EC50 values were calculated using the “drc” package in R 3.5.1. Software (Ritz and Streibig 2005; R Core Team 2019). For those Cd treatments with low mortality (the two lowest cadmium concentrations), the differences between the mean of the time to start activity of the control animals and this of animals at 48 h of cadmium exposure and at 168 h of post-exposure were compared with zero by means of a t-test (Field et al. 2012). This procedure allows to test if animals start activity faster (negative values) or slower (positive values) than control animals. The ‘t.test’ function was used in R (R Core Team 2019).

## Results and Discussion

The mean (± SD) (n = 12) physical-chemical parameters were: 17.9 ± 0.4ºC for water temperature, 9.19 ± 0.12 mg O_2_/l for dissolved oxygen, 352.4 ± 8.6 microS/cm for conductivity, and 8.09 ± 0.31 for pH. The mean actual (± SD) (n = 6) cadmium concentrations were 0.05 ± 0.01, 0.14 ± 0.03, 0.44 ± 0.06, and 1.34 ± 0.22 mg Cd/l for the four cadmium treatments (nominal concentrations of 0.05, 0.12, 0.5 and 1.5 mg Cd/L, respectively). Therefore, actual concentrations were very similar to nominal ones (0% of variation with the nominal concentration for the first treatment, 16.6% lower for the second treatment, 12% lower for the third treatment and 10.7% lower for the fourth treatment). Cadmium concentration in the control were less than 0.002 mg Cd/l (n = 6). Shell length of animals (mean ± SD) (n = 34) was 3.88 ± 0.16 mm.

**Fig. 1:**
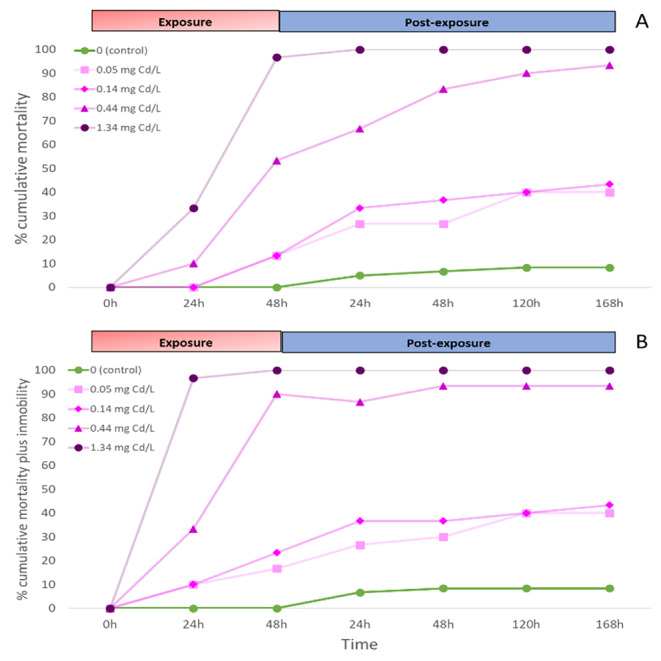
Percentage of cumulative mortality (A) and mortality plus immobility (B) of *Potamopyrgus antipodarum* for control and cadmium treatments (0.05, 0.14, 0.44 and 1.34 mg Cd/L) for each exposure (24 and 48 h) and post-exposure periods (24, 48, 120 and 168 h)

**Fig. 2:**
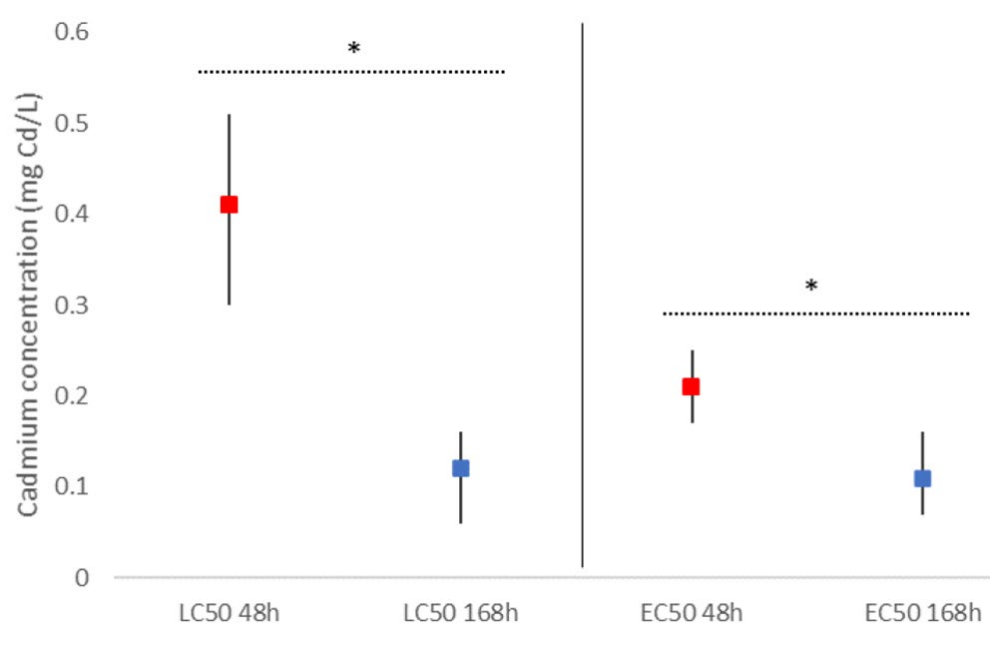
Lethal Concentrations 50 (LC50) and Effective Concentrations 50 (EC50) (mortality plus inactive animals) at 48 h of exposure and at 168 h of post-exposure for *Potamopyrgus antipodarum*. Squares represent the LC and EC, and the black lines represents the 95% confidence intervals. Asterisks show significant differences between the final exposure period (48 h) and the final of post-exposure period (168 h) for each endpoint (LC50 and EC50) (p < 0.05; overlap test)

The cumulative mortality in control was less than 10% (Fig. 1). During the exposure period, the two highest cadmium concentrations caused high cumulative mortality (97% at 1.34 mg Cd/L and 53% at 0.44 mg Cd/L) and high immobility plus mortality (100% at 1.34 mg Cd/L and 90% at 0.44 mg Cd/L) in *P. antipodarum* (Fig. 1 A and 1B). On the contrary, the two lowest concentrations had a relatively low effect during exposure to cadmium (13% for mortality in both concentrations, and 23% and 17% for cumulative immobility plus mortality) (Fig. 1 A and 1B). In the case of mortality, the post-exposure to cadmium caused an increase in mortality in all concentrations, with a marked increase in the three lowest concentrations (Fig. 1 A). A similar trend was found for the cumulative mortality plus immobility (Fig. 1B). The effect of post-exposure period to cadmium on LC50 and EC50 was significant (p < 0.05; Overlap test, Fig. 2). At 48 h of cadmium exposure, the LC50 was 0.41 (0.30–0.51) mg Cd/L and after the end of post-exposure the LC50 was 0.12 (0.06–0.16) mg Cd/L (Fig. 2). For the EC50 values, differences were significant but less marked (p < 0.05; Overlap test, Fig. 2), with a EC50 at 48 h of exposure of 0.21 (0.17–0.25) mg Cd/L and a EC50 at 168 h of 0.11 (0.07–0.16) mg Cd/L (Fig. 2). In general, during post-exposure periods, animals exposed to metals show an increase of mortality (Pascoe and Shazili 1986; Handy 1994; Schill et al. 2003; Zhao and Newman 2004, 2006; Hoang et al. 2007; Mebane et al. 2021). In our study this was especially marked in the highest concentrations. Therefore, mortality during post-exposure to toxicants should be included in the mortality endpoints (e.g., LC values) for a precise estimation of the real effects of toxicants (Zhao and Newman 2004). Models based on postexposure may enhance our predictive capabilities on the adverse effects of metals in field populations (Zhao and Newman 2004, 2006), which allows a better ecotoxicological risk assessment of toxicants.

**Fig. 3:**
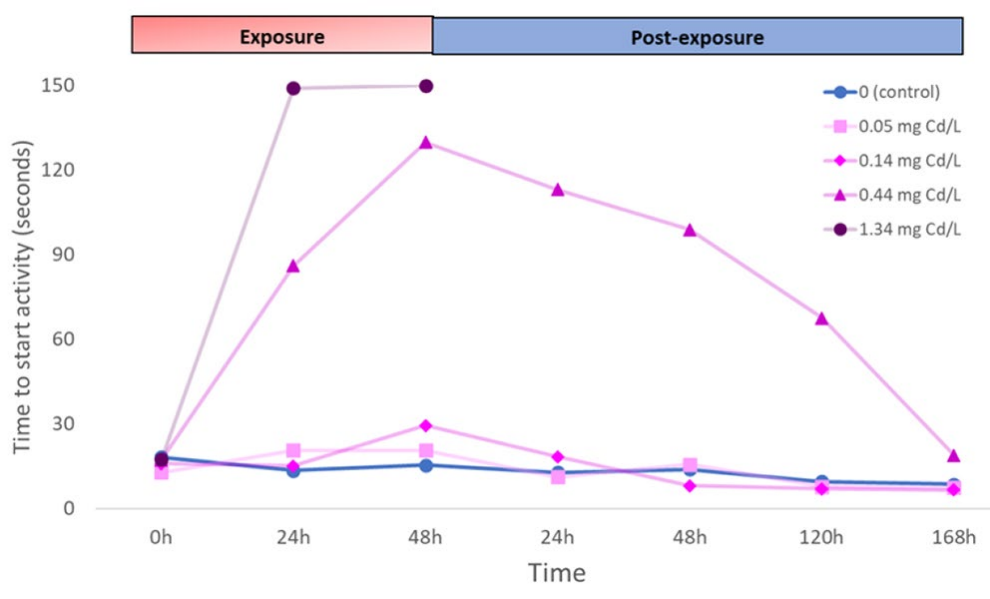
Time to start activity (in seconds) of *Potamopyrgus antipodarum* for control and each cadmium treatment (0.05, 0.14, 0.44 and 1.34 mg Cd/L) during the exposure and post-exposure periods. Standard deviations have been removed for clarity. Only active animals have been included

**Fig. 4:**
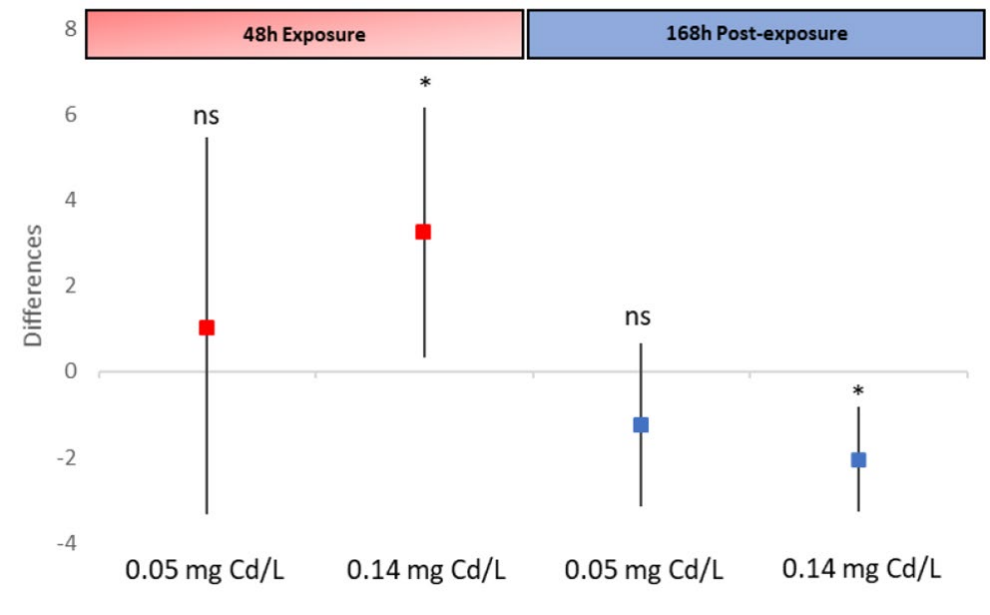
Mean (n = 17–18) differences (and 95% confidence intervals) between the time to start activity of each animal and the mean activity of control for 48 h of exposure and for 168 h of post-exposure to cadmium (0.05 and 0.14 mg Cd/L). Asterisks indicate significant differences with respect to zero for each cadmium concentration and exposure and post-exposure periods (p < 0.05; t-test). ns: no significant. Cadmium treatments of 0.44 and 1.34 mg Cd/L were not analyzed as the number of active animals was less than 3

During the post-exposure to cadmium, the time to start activity showed a clear recovery in surviving animals exposed to 0.44 mg Cd/L (Fig. 3). Under the lower cadmium concentrations (0.05 and 0.14 mg Cd/L), animals showed a trend similar to the control. The differences of time to start activity between cadmium treatments (0.05 and 0.14 mg Cd/L) and the mean control activity at the end of exposure (48 h) and at the end of post-exposure (168 h) are shown in Fig. 4. Differences were higher than cero at 48 h of exposure for 0.14 mg Cd/L, which means a lower activity than controls (p < 0.05; t-test, Fig. 4). Animals of this treatment at the end of the recovery period showed a higher activity than those of the control (p < 0.05; t-test, Fig. 4), which could indicate overexcitation caused by the exposure to cadmium. The 0.05 mg Cd/L treatment did not differ from the control in any time (p > 0.05; t-test, Fig. 4). Therefore, this concentration was safe to avoid behavioral impairments in *P. antipodarum*. This concentration was lower that the lower limit of the EC50 at 168 h of post-exposure to cadmium (0.07 mg Cd/L) (Fig. 2).

During cadmium exposure, animals may uptake a high amount of toxicant that subsequently cause lethal and sublethal effects on organisms during the post-exposure period (Pascoe and Shazili 1986; Wright and Welbourn 1994; Brent and Herricks 1998; Schill et al. 2003; Alonso and Valle-Torres 2018). A similar trend was also found for zinc (Mebane et al. 2021). The increase in mortality during the post-exposure period may be due to the damage on the detoxification mechanisms, reducing their effectiveness, and increasing the adverse effects of toxicants (Downs et al. 2001; Alonso and Camargo 2009; Holmstrup et al. 2010; Alonso and Valle-Torres 2018). During the exposure to stress, several physiological, biochemical, and behavioral adjustments are necessary to face stress and maintain homeostasis (Downs et al. 2001; Dao et al. 2013; Bertrand et al. 2017; Araujo and Blasco 2019; Fan et al. 2019). All of them produce a decrease in energy reserves, which can impair overall performance of organisms, including survival, growth, reproduction, and behavior (Calow 1991; Bryan et al. 1995; Brent and Herricks 1998; Dao et al. 2013; Bertrand et al. 2017; Fan et al. 2019). The high mortality during post-exposure may be due to exceeding the threshold of cadmium tolerance in the most sensitive individuals. The remaining tolerant animals may present efficient detoxification mechanisms, which can help to recover the behavior and avoid death.

The present study has shown that the inclusion of post-exposure in ecotoxicological bioassays allows a realistic assessment of the cadmium effects. The LC and EC values calculated with post-exposure effects showed a higher sensitivity of *P. antipodarum*, compared to exposure values. This fact should be taken into account for an adequate environmental risk assessment of toxicants. However, the activity showed a certain recovery in the animals that survived, which may indicate a certain cadmium detoxification in the individuals that were more tolerant to cadmium.
